# Validation of the effects of molecular marker polymorphisms in LcyE and CrtRB1 on provitamin A concentrations for 26 tropical maize populations

**DOI:** 10.1007/s00122-012-1987-3

**Published:** 2012-10-02

**Authors:** Raman Babu, Natalia Palacios Rojas, Shibin Gao, Jianbing Yan, Kevin Pixley

**Affiliations:** 1 CIMMYT, Apdo Postal 6–641, 06600 Mexico, D. F. Mexico; 2 Maize Research Institute, Sichuan Agricultural University, Ya’an, China; 3 National Key Laboratory of Crop Improvement, Huazhong Agricultural University, Wuhan, China

## Abstract

Vitamin A deficiency (VAD) compromises immune function and is the leading cause of preventable blindness in children in many developing countries. Biofortification, or breeding staple food crops that are rich in micronutrients, provides a sustainable way to fight VAD and other micronutrient malnutrition problems. Polymorphisms, with associated molecular markers, have recently been identified for two loci, LcyE (lycopene epsilon cyclase) and CrtRB1 (β-carotene hydroxylase 1) that govern critical steps in the carotenoid biosynthetic pathway in maize endosperm, thereby enabling the opportunity to integrate marker-assisted selection (MAS) into carotenoid breeding programs. We validated the effects of 3 polymorphisms (LcyE5′TE, LcyE3′Indel and CrtRB1-3′TE) in 26 diverse tropical genetic backgrounds. CrtRB1-3′TE had a two-ten fold effect on enhancing beta-carotene (BC) and total provitamin A (proA) content. Reduced-function, favorable polymorphisms within LcyE resulted in 0–30 % reduction in the ratio of alpha- to beta-branch carotenoids, and increase in proA content (sometimes statistically significant). CrtRB1-3′TE had large, significant effect on enhancing BC and total ProA content, irrespective of genetic constitution for LcyE5′TE. Genotypes with homozygous favorable CrtRB1-3′TE alleles had much less zeaxanthin and an average of 25 % less total carotenoid than other genotypes, suggesting that feedback inhibition may be reducing the total flux into the carotenoid pathway. Because this feedback inhibition was most pronounced in the homozygous favorable LcyE (reduced-function) genotypes, and because maximum total proA concentrations were achieved in genotypes with homozygous unfavorable or heterozygous LcyE, we recommend not selecting for both reduced-function genes in breeding programs. LcyE exhibited significant segregation distortion (SD) in all the eight, while CrtRB1 in five of eight digenic populations studied, with favorable alleles of both the genes frequently under-represented. MAS using markers reported herein can efficiently increase proA carotenoid concentration in maize.

**Electronic supplementary material:**

The online version of this article (doi:10.1007/s00122-012-1987-3) contains supplementary material, which is available to authorized users.

## Introduction

Vitamin A deficiency (VAD) is a health problem in more than half of all countries, resulting in visual impairment or blindness, and increased morbidity and mortality of at least 190 million preschool-age children and 19 million pregnant women, mostly in Africa and South Asia (WHO 2009). Both supplements and fortification of basic foods, for example sugar and milk, can help alleviate VAD, but the ideal and sustainable solution is consumption of adequate, nutritious diets. Unfortunately, poverty often results in chronic lack of access to well-balanced diets, and particularly to meat products and vegetables that are the best sources of many micronutrients, including vitamin A and provitamin A (proA) carotenoids (the precursors of vitamin A). As food prices increase, over-reliance on generally abundant, relatively inexpensive, widely traded, and easily stored staple grains often increases. Staple food grains are mainly rice in South Asia, wheat in Central Asia, and maize for most of sub-Saharan Africa. These are rich sources of energy, but they typically contain nutritionally inadequate quantities of micronutrients, including proA carotenoids (see Nuss and Tanumihardjo (2010) for a discussion of the nutritional qualities of maize). Average per capita consumption of maize in Zambia, Malawi, Lesotho, South Africa and Mexico, for example, is more than 100 kg per year, providing 30–60 % of both calories and protein for these consumers (FAO Stat, as cited by Atlin et al. 2011).

Biofortification is the breeding of staple food crops to increase micronutrient density (Bouis and Welch 2010; Pfeiffer and McClafferty 2007). Graham et al. (2001) suggested that because of the widespread consumption of staple crops, biofortification may be an effective and sustainable way of addressing micronutrient malnutrition. The proponents of biofortification include more than nutritionists and agriculturalists. From an economic point of view, investments in biofortification are justified as a complementary strategy to supplementation and fortification, particularly suited to rural or remote areas where other approaches may have incomplete coverage (Copenhagen Consensus 2008; Qaim et al. 2007). HarvestPlus is a CGIAR (Consultative Group on International Agricultural Research) generation challenge program that focuses on three critical micronutrients that are recognized by the World Health Organization (WHO) as most limited in diets: iron, zinc, and proA. HarvestPlus set preliminary minimum target level of 15 μg/g for proA for maize, based on gross assumptions of daily intake (400 g for adults and 200 g for 4–6 years old children), bioavailability ratio (12:1 μg to retinol activity equivalent) and retention after processing (50 %) (Bouis et al. 2011).

One of the biggest challenges to breeding proA biofortified maize is the low throughput and high cost of quantifying carotenoid content in maize grain. High performance liquid chromatography (HPLC) currently costs USA $50–$100 or more per sample, and while ultra-performance liquid chromatography (UPLC) is a very good alternative to HPLC because its costs for reagents are lower, and throughput is three times that of HPLC, neither HPLC nor UPLC enable efficient and affordable analysis of the many thousands of samples required each year by a breeding program (Palacios Rojas, in preparation). Although the visible light range (400–1,100 nm) is important for predicting carotenoid content in maize grain, calibration curves for estimating carotenoid concentrations using near infra-red reflectance spectroscopy (NIRS) have been successful for estimating the major carotenoids (lutein and zeaxanthin) and total carotenoid, but not for proA carotenoid concentrations (Berardo et al. 2009).

**Fig. 1:**
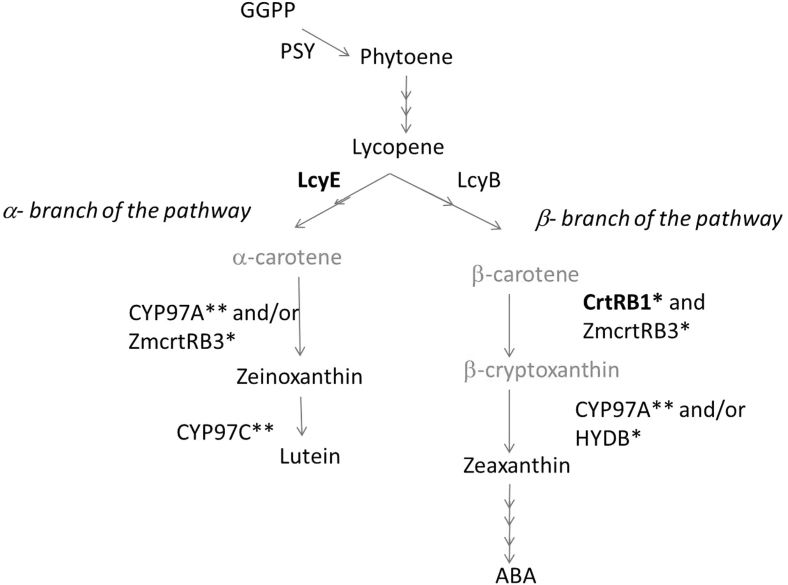
Carotenoid byosinthetic pathway. Provitamin A carotenoids are highlighted in *orange*. Enzymes with allelic polymorphism studied here are in *bold*. *Single asterisk* indicates non-heme di-iron enzymes; *double asterisks* indicate cytochrome p450 enzymes. *GGPP* geranyl geranyl diphosphate, *PSY* phytoene synthase, *LCY*β β-cyclase, *LCY*ε ε-cyclase, *ABA* abscisic acid. Based on Meier et al. (2011); Wurtzel et al. (2012) and Zhou et al. (2012)

Marker-assisted selection (MAS) complements conventional breeding through use of inexpensive DNA markers that are tightly linked to a target locus or loci. Identifying the causal loci is an important pre-requisite to enable MAS in breeding programs. The carotenoid metabolic pathway has been well researched in model species and key genes governing critical steps have been identified (Giuliano et al. 2008) (Fig. 1). In maize, three genes have been proposed to play crucial roles in the final accumulation of proA carotenoids in the grain. Phytoene synthase1 (*Y1* or *Psy1*) catalyses the first committed step in the pathway leading to formation of phytoene from geranylgeranyl diphosphate and is primarily responsible for the shift from white to yellow maize (Li et al. 2009). Two genes, lycopene epsilon cyclase (LcyE) and beta-carotene hydroxylase 1 (CrtRB1) have been shown to regulate the accumulation of proA compounds. LcyE converts lycopene into zeta-carotene and eventually to alpha-carotene through the action of other associated genes. Naturally existing mutant alleles of LcyE with reduced functionality have been identified that apportion more lycopene into the beta branch of the pathway, thereby enhancing the flux toward proA compounds (Fig. 1) (Harjes et al. 2008). CrtRB1 is a hydroxylase gene that converts BC into beta-cryptoxanthin (BCX), whose proA activity is theoretically only half that of BC. Natural genetic variation for CrtRB1 has recently been discovered that results in the retention of more BC in the maize endosperm (Yan et al. 2010).

Molecular markers based on functional polymorphisms within Psy1, LcyE and CrtRB1, and perhaps other genes as well, hold great potential for accelerated and resource-efficient development of proA enriched lines. Markers located within the target genes offer efficient means of tracking the favorable alleles in backcross or pedigree breeding programs. However, genetic background in which these favorable alleles reside, population size, nature of gene action (additive or epistatic), trait heritability and marker–trait relationships influence the effectiveness of such MAS in routine breeding programs.

Though the potential applications of MAS are attractive for selecting natural variants of large-effect carotenoid genes, it is rarely a straight-forward exercise in a practical breeding program. Implementation of MAS for LcyE and CrtRB1 in CIMMYT’s HarvestPlus maize proA carotenoid breeding program required addressing several operational constraints such as (1) markers that were diagnostic of causative polymorphisms within LcyE, developed based on temperate maize germplasm, were frequently found not to be reproducible in tropical and sub-tropical genetic backgrounds; (2) the PCR programs were sensitive to the machine and cycling conditions used and resulted in frequent amplification failures, indicating that these genes may reside in PCR-recalcitrant genomic regions and hence may require different assay procedures for efficient screening; (3) the effects of LcyE and CrtRB1 in diverse genetic backgrounds, especially in tropical and sub-tropical germplasm are not well established; and (4) both LcyE and CrtRB1 showed severe segregation distortion in the few populations studied at CIMMYT, resulting in very low favorable allele frequency compared to their Mendelian expectations.

The objectives of this investigation were to (1) evaluate the phenotypic effects of different functional polymorphisms within LcyE and CrtRB1, alone as well as in combination, in diverse tropical and sub-tropical germplasm; and (2) study the extent of segregation distortion for these two genes in diverse crosses to enable making informed decisions about effective population size in breeding programs.

## Materials and methods

### Plant materials/population development

Unless otherwise stated, the germplasm used for this study is from CIMMYT’s proA biofortification of maize breeding program funded by HarvestPlus (see supplementary materials, Table S1). The functional polymorphism classes for LcyE and CrtRB1 are listed in Table 1. All allelic nomenclature for LcyE corresponds to that reported by Harjes et al. (2008), while nomenclature for CrtRB1 is as reported by Yan et al. (2010). It may assist the reader to recall that the unfavorable allele for both LcyE and CrtRB1 is always named ‘allele 2’, while all other alleles are considered favorable for their respective gene.

**Table 1:** Functional polymorphism classes for LcyE and CrtRB1 that were tested in the present study

Gene	Allele	Nomenclature	Description
LcyE	LcyE-5′TE	2	Unfavorable
		4	Favorable
	LcyE-3′Indel	2	Unfavorable
		1	Favorable
CrtRB1	CrtRB1-3′TE	2	Unfavorable
		1	Favorable

The nomenclature for different functional polymorphism assets were according to Harjes et al. (2008) for LcyE and Yan et al. (2010) for CrtRB1

Five F_2_ populations (L1–L5) were developed for use in validating the effects of three functional polymorphisms (two favorable alleles of LcyE-5′TE and one of LcyE-3′Indel) within LcyE. CIMMYT maize line 297 (CML297), a late-maturing, lowland tropical line, provided the favorable ‘allele 4’ for LcyE-5′TE in populations L2 and L3, and ‘allele 1’ for LcyE-3′Indel in population L4. In population L1, Carotenoid Syn3-FS11-4-3-B–B–B, a tropicalized line derived from a temperate synthetic population obtained from Dr. Torbert Rocheford, University of Illinois, provided the favorable ‘allele 4’ for LcyE-5′TE. Among more than 100 lines surveyed at CIMMYT for different alleles of LcyE-5′TE, only Hi27 was found to have ‘allele 1’, and population L5 was its cross with CML328. In L5, both CML297 and P72c1xCML297 × CL02410-3-1-1-B-B had favorable ‘allele 4’ but differed for LcyE-3′Indel polymorphism, thereby allowing us to study the effect of favorable allele (‘allele 1’) of LcyE-3′indel in the favorable allele background of LcyE-5′TE. All five populations L1–L5 were homozygous for the wild-type (unfavorable) allele of CrtRB1.

For validating the effect of the CrtRB1-3′TE polymorphism, individuals with contrasting genotypes were identified in 15 F_3_:F_4_ populations (H1–H15), representing 5 different genetic backgrounds (Table S2). An intermediate maturity tropical line, (KU1409/DE3/KU1409)S2-18-2-B, from the International Institute of Tropical Agriculture (IITA), served as the donor of the favorable ‘allele 1’ for CrtRB1-3′TE polymorphism in all the crosses; however, this line is a backcross-derived line in which the temperate line ‘DE3’ provided the favorable allele. LcyE background of these populations was homozygous favorable ‘allele 4’ for eight populations, homozygous unfavorable ‘allele 1’ for five populations, and heterozygous or segregating in two populations (Table S2).

Six digenic F_2_ populations (Digenic 1–6) were developed for studying the combined and interaction effects of LcyE and CrtRB1 functional polymorphisms on final accumulation of proA-related compounds. The tropical line from IITA provided the favorable ‘allele 1’ for CrtRB1-3′TE polymorphism in all crosses, while six different lines contributed the favorable allele of LcyE-5’TE.

Population Digenic 1–6, plus two more digenic populations derived from temperate germplasm (KUI3 × B77 and KUI3 × SC55), were used for segregation distortion (SD) assessment.

### Genotyping for LcyE and CrtRB1 polymorphisms

Genomic DNA was extracted from leaves and seeds using CTAB-based protocol (CIMMYT Applied Genetics Laboratory, 2003, Lu et al. 2010 and Gao et al. 2008). The primer combinations for LcyE-5′TE and LcyE-3′indel were slightly changed from Harjes et al. (2008), in order to get reproducible results. We used the following set of primers—LcyE-5′TE–F: AAGCAGGGAAGACATTCCAG and LcyE-5′TE-R: GAGAGGGAGACGACGAGACAC for LcyE-5′TE; LcyE-3′Indel-F: ACCCGTACGTCGTTCATCTC and LcyE-3′Indel-R: ACCCTGCGTGGTCTCAAC for the LcyE-3′Indel polymorphism. For the CrtRB1-3′TE polymorphism, we used the 3-primer cocktail as reported by Yan et al. (2010) without any modification. The PCR and gel-electrophoresis conditions were followed according to Harjes et al. (2008) and Yan et al. (2010).

### Carotenoid phenotyping

For populations L1–L5, individual seeds were genotyped using a non-destructive DNA extraction and genotyping system (Gao et al. 2008). For each genotype class within each population, 25–50 seeds were bulked and phenotyped for carotenoids using HPLC. For other populations, seed from ears harvested from plants of known genotype class were used for phenotyping.

Carotenoid extraction was performed as described by Pixley et al. (2011). Briefly, carotenoids were released from finely ground dried maize grain samples by adding ethanol, samples were then saponified, followed by carotenoid extraction using hexane prior to separation and quantification using HPLC with a 30C column attached to a YMC C30 filter insert. A multi-wavelength detector set at 450 nm was used, and data were collected and processed using Waters Millennium 2010 software (Waters Chromatography, Milford, MA, USA). Lutein, zeaxanthin, β-cryptoxanthin (BCX) and all-trans-β-carotene (BC) were identified through their characteristic spectra and comparison of their retention times with known standard solutions.

Total ProA content (μg g^−1^ of dry matter) was calculated for each sample as the sum of BC plus one-half of BCX. Ratio of α- to β-branch carotenoids was estimated as: [lutein/(zeaxanthin + BC + BCX)].

### Statistical analyses

Segregation distortion was assessed by Chi-square tests of the deviation from the expected Mendelian ratio (1:2:1 for F_2_ populations) using the two loci independent marker data. A mixed effects model with fixed effects of genotype classes and their interactions, and random effects of populations was used to analyze the carotenoid data. Data were analyzed using PROC MIXED of SAS, assuming that the error variance was normally distributed with mean 0 and that the error and the fixed effects were uncorrelated. Mean for treatment effects was estimated using PROC MIXED and REML (Restricted/Residual maximum likelihood), in which estimators are obtained not from maximizing the whole likelihood function, but only that part which is invariant to the fixed effects part of the linear model. LSMEANS option was used to compute the least squares mean of fixed effects. The ADJUST option was used to request a multiple comparison adjustment to the *p* values for pair-wise comparisons of mean.

## Results

### Effects of LcyE polymorphisms

Three different functional polymorphisms of LcyE (LcyE5′TE and LcyE3′Indel) across the five populations L1–L5 did not result in statistically significant differences either for ratio of α- to β-branch carotenoids or for total ProA carotenoids concentration (Table 2). Although two (L1 and L2) of the three populations with the favorable ‘allele 4’ had significantly more carotenoids in the β- than α-branch (Table 3), this effect on ratio was not statistically significant when analyzed across the populations (Table 2). The LcyE alleles also did not significantly affect total proA carotenoid concentration of the genotypes (Table 3). The second favorable LcyE-5′TE allele (‘allele 1’) also resulted in no significant differences for total proA concentration and for ratio of α- to β-branch carotenoids (Population L4, Table 3). Finally, the favorable ‘allele 1’ for the LcyE-3′Indel polymorphism (population L5), hypothesize that in the maize kernel, feedback inhibitionsignificantly reduced the ratio of α- to β-branch carotenoids from 0.29 to 0.20 and increased the total proA carotenoid concentration from 2.59 to 2.77 μg g^−1^ (Table 3), but we hypothesize that such changes are too small to be of biological significance.

**Table 2:** Tests of fixed effects for three genotype classes of LcyE in 5 and CrtRB1 in 15 mono-genic segregating populations and their 9 interaction classes in 6 digenic segregating populations for LUT, ZEA, BCX, BC, ProA, and Ratio

Effect	*df*	LUT	ZEA	BCX	BC	ProA	Ratio
		*F* val	*F* val	*F* val	*F* val	*F* val	*F* val
LcyE genotype	12	1.92	0.05	0.03	0.00	0.01	0.78
CrtRB1 genotype	42	1.25	7.73*	0.39	18.71**	13.59**	1.01
CrtRB1 and LcyE interaction	72	7.51**	11.17**	18.93**	20.97**	16.48**	12.20**

This is not an ANOVA table; each row in this table is extracted from ANOVA for different sets of data/populations

ProA = BC + (0.5 × BCX)

Ratio = LUT/(ZEA + BCX + BC)

*F* value followed by *, ** or no symbol indicates significance at *P* < 0.01, *P* < 0.001, and not significant, respectively

*LUT* Lutein, *ZEA* zeaxanthin, *BCX* betacryptopxanthin, *BC* beta-carotene, *ProA* total provitamin A carotenoids, *Ratio* ratio of α- to β-branch carotenoids

**Table 3:** Effect of various favorable alleles for different functional polymorphisms of LcyE in five mono-genic segregating populations

Polymorphism	Population	Pedigree	Genotype class	No. of kernels	LUT	ZEA	BCX	BC	ProA	Ratio
LcyE-5′TE	L1-P1	CML486	“2” (U)	25	11.4 ± 0.6	21.7 ± 1.9	9.1 ± 0.3	0.76 ± 0.04	5.04 ± 0.41	0.36 ± 0.01
	L1-P2	Carotenoid Syn3-FS11-4-3-B–B–B	“4” (F)	25	5.9 ± 0.4	14.3 ± 2.1	13.0 ± 1.2	1.10 ± 0.06	8.1 ± 0.70	0.21 ± 0.02
	L1-F2	CML486/Carotenoid Syn3-FS11-4-3-B–B–B	“2”	57	4.5 ± 0.2	9.0 ± 0.8	6.1 ± 0.5	0.69 ± 0.02	7.51 ± 0.74	0.28 ± 0.00
			“4”	33	2.4 ± 0.3	9.01 ± 1.8	6.7 ± 0.8	0.78 ± 0.04	8.28 ± 1.17	0.14 ± 0.00
			“H”	79	2.4 ± 0.4	8.01 ± 1.4	6.72 ± 1.1	0.68 ± 0.09	8.08 ± 0.50	0.16 ± 0.03
LcyE-5′TE	L2-P1	DRB-F2-60-1-1-1-BB/[BETASYN]BC1-9-#-B-B	“2” (U)	25	0.7 ± 0.05	4.1 ± 0.3	3.8 ± 0.2	0.56 ± 0.04	2.22 ± 0.37	0.15 ± 0.01
	L2-P2	CML297	“4” (F)	25	2.9 ± 0.03	10.4 ± 0.4	8.3 ± 0.4	0.57 ± 0.03	5.12 ± 0.51	0.09 ± 0.02
	L2-F2	CML-297-B/DRB-F2-60-1-1-1-BB/[BETASYN]BC1-9-#-B	“2”	42	1.0 ± 0.05	3.7 ± 0.6	4.4 ± 0.5	0.59 ± 0.05	5.63 ± 0.34	0.12 ± 0.03
			“4”	46	0.7 ± 0.05	3.8 ± 0.4	3.3 ± 0.6	0.38 ± 0.02	4.13 ± 0.39	0.09 ± 0.01
			“H”	85	1.3 ± 0.12	4.0 ± 0.8	4.2 ± 0.8	0.51 ± 0.06	5.30 ± 0.38	0.16 ± 0.01
LcyE-5′TE	L3-P1	MAS[206/312]-23-2-1-1-B–B–B/[BETASYN]BC1-11-3-1-#-B	“2” (U)	25	1.5 ± 0.1	2.4 ± 0.4	2.5 ± 0.2	0.96 ± 0.05	2.4 ± 0.26	0.26 ± 0.01
	L3-P2	CML297	“4” (F)	25	0.6 ± 0.05	4.6 ± 0.3	2.4 ± 0.1	0.47 ± 0.06	1.68 ± 0.16	0.09 ± 0.02
	L3-F2	MAS[206/312]-23-2-1-1-B-B-B/[BETASYN]BC1-11-3-1-#-B/CML297	“2”	29	1.1 ± 0.3	3.1 ± 0.2	2.6 ± 0.3	0.86 ± 0.1	2.18 ± 0.16	0.17 ± 0.02
			“4”	19	0.7 ± 0.06	3.0 ± 0.5	2.4 ± 0.4	0.96 ± 0.14	2.20 ± 0.07	0.11 ± 0.02
			“H”	28	0.7 ± 0.05	2.3 ± 0.4	2.3 ± 0.4	0.91 ± 0.08	2.09 ± 0.24	0.12 ± 0.01
LcyE-5′TE	L4-F2	Hi27XCML328-F2	“2” (U)	54	0.9 ± 0.12	12.2 ± 1.6	4.2 ± 0.3	1.10 ± 0.2	3.21 ± 0.42	0.05 ± 0.01
			“1” (F)	35	0.6 ± 0.08	11.8 ± 0.9	4.5 ± 0.2	1.16 ± 0.1	3.44 ± 0.53	0.03 ± 0.01
			“H”	32	0.5 ± 0.05	10.4 ± 1.8	4.9 ± 0.7	1.12 ± 0.1	3.57 ± 0.50	0.03 ± 0.01
LcyE-3′Indel	L5-P1	P72c1xCML-297 × CL-02410-3-1-1-B–B	“2” (U)	25	6.0 ± 0.5	10.2 ± 0.9	3.1 ± 0.1	0.39 ± 0.02	1.94 ± 0.00	0.44 ± 0.03
	L5-P2	CML297	“1” (F)	25	0.6 ± 0.08	4.6 ± 0.3	2.4 ± 0.1	0.47 ± 0.05	1.68 ± 0.16	0.09 ± 0.02
	L5-F2	P72c1xCML-297 × CL-02410-3-1-1-B/CML-297-B–B	“2”	23	3.8 ± 0.2	9.2 ± 1.2	3.1 ± 0.4	1.00 ± 0.08	2.59 ± 0.04	0.29 ± 0.01
			“1”	24	2.9 ± 0.4	10.1 ± 0.6	3.5 ± 0.2	1.03 ± 0.1	2.77 ± 0.05	0.20 ± 0.00
			“H”	42	3.1 ± 0.3	10.3 ± 0.4	3.71 ± 0.5	1.10 ± 0.05	2.96 ± 0.37	0.21 ± 0.01

Concentrations are expressed as μg g^−1^ dry weight (DW). Parental lines (P1 and P2); segregating populations (F2)

*LUT* Lutein, *ZEA* zeaxanthin, *BCX* betacryptopxanthin, *BC* beta-carotene, *ProA* total provitamin A carotenoids, *Ratio* ratio of α- to β-branch carotenoids

### Effects of the CrtRB1-3′TE polymorphism

Least square mean and tests of fixed effects based on combined analysis of populations H1–H15 for CrtRB1 are presented in Table 4, while mean for different genotype classes within each of the 15 population is presented in Table S2. ‘Allele 1’ of CrtRB1-3′TE had significant, two-ten fold effect over ‘allele 2’ for BC and total proA carotenoid concentrations in the 15 populations. While ‘allele 1’ had no effect on BCX content, it significantly reduced zeaxanthin content for 14 of the 15 populations. Across the 15 populations, ‘allele 1’ of CrtRB1 was significantly associated with enhanced BC content and total proA concentration (Table 4), resulting on average in 3.8-times more BC and 2.4-times the proA in homozygous favorable relative to homozygous unfavorable ‘allele 2’ genotype. The heterozygous CrtRB1 genotype resulted, on average, in 1.9-times the BC and 1.5-times more proA than the homozygous unfavorable. Stated differently, the heterozygotes achieved about one-third of the total effect achieved by the homozygous favorable genotype for both BC and proA, suggesting partially recessive gene action, on average.

**Table 4:** Least square mean and tests of fixed effects for individual and contrast comparisons of CrtRB1 genotypes for different carotenoid compounds across 15 segregating populations representing 5 distinct genetic backgrounds

Individual and contrast effects of CrtRB1 genotypes	LUT	ZEA	BCX	BC	ProA
	Mean ± SE; *P* > |*t*|	Mean ± SE; *P* > |*t*|	Mean ± SE; *P* > |*t*|	Mean ± SE; *P* > |*t*|	Mean ± SE; *P* > |*t*|
HH (“1”)	10.9 ± 1.6	10.6 ± 2.9**	6.8 ± 0.7**	13.4 ± 1.1**	16.8 ± 1.3**
Hh (“H”)	14.5 ± 1.6	19.2 ± 2.9**	7.0 ± 0.7**	6.8 ± 1.1**	10.6 ± 1.3**
hh (“2”)	12.4 ± 1.6	26.6 ± 2.9**	7.6 ± 0.7**	3.5 ± 1.1*	7.0 ± 1.3**
HH (“1”) versus Hh (“H”)	3.6 ± 2.3	−8.7 ± 4.1*	0.2 ± 0.09^NS^	6.6 ± 1.7**	6.2 ± 1.9*
HH (“1”) versus hh (“2”)	1.5 ± 2.3	−16.1 ± 4.1**	0.8 ± 0.09^NS^	9.9 ± 1.7**	9.8 ± 1.9**
Hh (“H”) versus hh (“2”)	2.0 ± 2.3	−7.4 ± 4.1^NS^	0.6 ± 0.09^NS^	3.3 ± 1.7*	3.7 ± 1.9^NS^

*LUT* Lutein, *ZEA* Zeaxanthin, *BCX* Betacryptopxanthin, *BC* Beta-carotene, *ProA* total provitamin A carotenoids, *NS* not significant

ProA = BC + (0.5 × BCX)

* Significant at *P* < 0.05

** Significant at *P* < 0.01

Several interesting results became apparent when comparing the effects of CrtRB1 alleles separately for the eight populations with unfavorable ‘allele 2’ LcyE background and the five populations with favorable ‘allele 4’ of LcyE. Across the homozygous unfavorable ‘allele 2’ LcyE populations, the homozygous favorable ‘allele 1’ of CrtRB1 resulted in 4.2-times the BC and twice the ProA as compared to homozygous unfavorable ‘allele 2’ of CrtRB1. On the other hand, in populations with homozygous favorable ‘allele 4’ LcyE background, the homozygous favorable ‘allele 1’ of CrtRB1 resulted in only 1.2-times the BC and 0.6-times the ProA as compared to homozygous unfavorable ‘allele 2’ of CrtRB1. Although the comparison is among, instead of within populations, and thus may be confounded by differences in genetic backgrounds, the digenic homozygous favorable genotypes (‘allele 4’ LcyE with ‘allele 1’ CrtRB1) had on average 3.5-times the BC (10.2 vs. 1.9 μg g^−1^) and 2.2-times the proA concentration (13.8 vs. 6.4 μg g^−1^) relative to the digenic homozygous unfavorable genotypes (Table S2).

The homozygous favorable LcyE ‘allele 4’, in combination with the homozygous favorable CrtRB1 ‘allele 1’ resulted in an average of 25 % reduction in total carotenoid concentration (lutein + zeaxanthin + BC + BCX) relative to when it combined with the homozygous unfavorable ‘allele 2’ of CrtRB1. In contrast, combining the homozygous unfavorable LcyE ‘allele 2’ with homozygous favorable CrtRB1 ‘allele 1’ resulted in (probably insignificant) 7 % reduction in total carotenoid concentration relative to its combination with the homozygous unfavorable ‘allele 2’ of CrtRB1. Across the 15 populations H1–H15, that is, across all LcyE genotypic backgrounds, the homozygous favorable ‘allele 1’ resulted in a 21 % reduction in total carotenoid concentration relative to the unfavorable ‘allele 2’ of CrtRB1. The heterozygous CrtRB1 genotypes were most similar to wild-type (no reduction in total carotenoids) when in the unfavorable (wild-type) LcyE background, but were similar to homozygous favorable CrtRB1 (22 % reduction in total cartotenoid concentration) when in the favorable LcyE ‘allele 4’ background.

The highest total proA concentration, 19.2 μg g^−1^ on average, was achieved by populations (*n* = 7) with the homozygous unfavorable LcyE ‘allelle 2’ combined with homozygous favorable CrtRB1 ‘allele 1’ (ll_HH), followed by 13.8, 12.3 and 10.3 μg g^−1^ for populations (*n* = 5, 5 and 7, respectively) with LL_HH, LL_Hh and ll_Hh.

### Combined effects of LcyE-5′TE and CrtRB1-3′TE polymorphisms

The carotenoid profile of seeds belonging to nine genotype classes resulting from segregation of two alleles each for LcyE and CrtRB1 within six F_2_ populations was phenotyped by HPLC (Table 5). In contrast to results for populations L1–L5, the homozygous favorable LcyE ‘allele 4’ reduced lutein (≈60 %) and increased zeaxanthin (≈40 %), resulting in average reduction of 75 % in the ratio of α- to β-branch carotenoids, from 0.77 to 0.19 (Table 5). This shift in the flux through the carotenoid pathway resulted in 20–30 % increases in BC, BCX and total proA concentrations. The heterozygous genotypes for LcyE were statistically similar to the homozygous favorable (LL), indicating partial dominance mode of action (Table 5).

**Table 5:** Least square mean and tests of fixed effects for nine interaction classes of LcyE and CrtRB1

CrtRB1 and LcyE interaction class	Genotype	LUT	ZEA	BCX	BC	ProA	Ratio
		Mean ± SE; *P* > *F*	Mean ± SE; *P* > *F*	Mean ± SE; *P* > *F*	Mean ± SE; *P* > *F*	Mean ± SE; *P* > *F*	Mean ± SE; *P* > *F*
1	LL_HH	4.4 ± 1.3**	7.6 ± 1.7**	1.2 ± 0.3**	7.7 ± 0.6**	8.4 ± 0.5**	0.25 ± 0.09*
2	LL_Hh	3.9 ± 1.4*	15.9 ± 1.9**	2.7 ± 0.3**	4.5 ± 0.7**	5.9 ± 0.6**	0.16 ± 0.10 NS
3	LL_hh	3.9 ± 1.3*	16.5 ± 1.6**	4.4 ± 0.3**	1.6 ± 0.6*	3.8 ± 0.5**	0.16 ± 0.09 NS
4	ll_HH	9.9 ± 1.1**	2.9 ± 1.5 NS	0.4 ± 0.2 NS	7.1 ± 0.5**	7.3 ± 0.4**	0.98 ± 0.08**
5	ll_Hh	12.1 ± 0.9**	11.0 ± 1.2**	2.7 ± 0.2**	2.8 ± 0.4**	4.2 ± 0.4**	0.83 ± 0.07**
6	ll_hh	9.3 ± 1.0**	14.9 ± 1.3**	3.1 ± 0.2**	1.6 ± 0.5*	3.2 ± 0.4**	0.51 ± 0.07**
7	Ll_HH	5.3 ± 1.4**	3.9 ± 1.9*	0.9 ± 0.3*	9.0 ± 0.7**	9.5 ± 0.6**	0.40 ± 0.10**
8	Ll_Hh	5.3 ± 1.0**	14.3 ± 1.3**	3.1 ± 0.2**	3.8 ± 0.5**	5.4 ± 0.4**	0.27 ± 0.07**
9	Ll_hh	5.9 ± 1.4**	18.6 ± 1.9**	3.7 ± 0.3**	2.0 ± 0.7*	3.9 ± 0.6**	0.24 ± 0.11*
Mean (1, 2, 3)	LL–	4.07^a^	13.33^a^	2.77^a^	4.60^a^	6.03^a^	0.19^a^
Mean (4, 5, 6)	Ll–	5.50^a^	12.27^ab^	2.57^ab^	4.93^a^	6.27^a^	0.30^a^
Mean (7, 8, 9)	ll–	10.43^b^	9.60^b^	2.07^b^	3.83^a^	4.90^b^	0.77^b^
Mean (1, 4, 7)	–HH	6.53^a^	4.80^a^	0.83^a^	7.93^a^	8.40^a^	0.54^a^
Mean (2, 5, 8)	–Hh	7.10^a^	13.73^b^	2.83^b^	3.70^b^	5.17^b^	0.42^ab^
Mean (3, 6, 9)	–hh	6.37^a^	16.67^b^	3.73^c^	1.73^c^	3.63^c^	0.30^b^

*LUT* Lutein, *ZEA* zeaxanthin, *BCX* betacryptopxanthin, *BC* beta-carotene, *ProA* total provitamin A carotenoids, *Ratio* ratio of α- to β-branch carotenoids, *NS* not significant

*^,^ ** Significant at *P* < 0.05, *P* < 0.01, respectively

^a, b, c and ab^ Indicate DMRT significance

LS mean with same letter is not significantly different

The favorable CrtRB1 ‘allele 1’ had no effect on lutein, but greatly reduced the concentration of zeaxanthin in grain, resulting in an increase in the ratio of α- to β-branch carotenoids, from 0.30 to 0.54 (Table 5). The homozygous favorable (HH) had around one-fourth the BCX and 4.6-times the BC, resulting in 2.3-times more proA, on average, than the homozygous unfavorable genotypes (hh). The heterozygous genotypes (Hh) were statistically same or closest to the unfavorable (hh) homozygotes for zeaxanthin and BCX concentrations, whereas BC and ProA concentrations for the Hh were intermediate to the homozygote types.

**Fig. 2:**
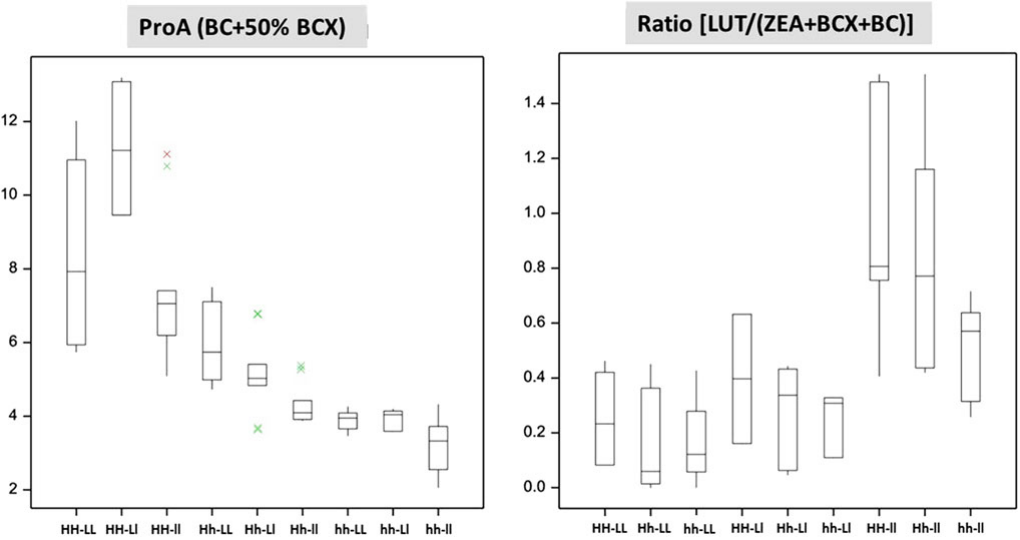
Combined effect of CrtRB1-3′TE and LcyE-5′TE on ProA and ratio based on six F_2_ populations. *H* refers to favorable allele 1 and *h* to unfavorable allele 2 of CrtRB1-3′TE polymorphism; similarly, *L* refers to favorable allele 4 and *l* to unfavorable allele 2 of LcyE-5′TE polymorphism

Across populations Digenic 1–6, the double favorable homozygote class (LL_HH) had more BC (3.8 times) and ProA (1.6 times), had lower ratio of α- to β-branch carotenoids (51 %), and had less BCX (61 %), zeaxanthin (49 %) and lutein (53 %) than the double unfavorable homozygotes (ll_hh). However, similar to noted above for populations H1–H15, populations Digenic 1–6 also experienced a pronounced average reduction in total carotenoid concentration when the homozygous favorable CrtRB1 ‘allele 1’ was present (Fig. 2). This decrease in total carotenoid concentration relative to the homozygous unfavorable CrtRB1 ‘allele 2’ was 30 % across all LcyE genotypic backgrounds, and 28 % for the double homozygous favorable (LcyE ‘allele 4’ with CrtRB1 ‘allele 1’) relative to double homozygous unfavorable genotypes (Table 5). The heterozygous were similar to homozygous unfavorable CrtRB1 genotypes having almost no reduction in total carotenoid concentration. The three LcyE genotypes, when considered across CrtRB1 genotypes, had less than 5 % variance for total carotenoid concentration.

Of the nine genotype classes, the top three in terms of BC and ProA concentrations were Ll_HH, LL_HH and ll_HH, confirming the pronounced effect of the favorable ‘allele 1’ of CrtRB1, irrespective of LcyE genetic constitution.

### Segregation distortion (SD) for LcyE and CrtRB1

Significant SD was observed for LcyE in all eight, and for CrtRB1 in five of the eight F_2_ populations (Table 6). The extent of SD was severe for LcyE and mostly moderate for CrtRB1, and appeared to be influenced by genetic background. Unfortunately, SD was skewed toward the unfavorable and/or heterozygote allele genotypes, and the favorable allele was therefore under-represented for both LcyE and CrtRB1 in most of the populations.

**Table 6:** Segregation distortion for LcyE and CrtRB1 as observed in 8 F_2_ populations

Population (F2)	Number of seeds identified			Chi-square	Number of seeds identified			Chi-square
	LL (“4”)	Ll (“H”)	ll (“2”)		HH (“1”)	Hh (“H”)	hh (“2”)	
Digenic-1	26	202	125	62.9**	101	153	115	11.8**
Digenic-2	12	82	274	486.2**	75	94	160	104.3**
Digenic-3	42	29	188	320.6**	79	111	69	6.1*
Digenic-4	10	8	190	488.9**	89	166	104	3.3 ^**ns**^
Digenic-5	45	44	97	80.7**	45	59	82	39.6**
Digenic-6	56	215	98	19.6**	90	191	77	2.6 ^**ns**^
Digenic-7	84	138	126	25.0**	68	164	116	14.4**
Digenic-8	62	124	127	40.5**	83	151	79	0.5 ^**ns**^
Across all populations	337	842	1225	871.6**	630	1089	802	69.5**

See Table S1 for complete pedigrees for populations Digenic-1 to Digenic-8

*significant at P <0.05; **significant at P <0.01 respectively

ns Not Significant

## Discussion

Our results contrast with the Harjes et al. (2008) report of significant reduction in lutein content and concomitant increase in total proA concentration for the LcyE-5′TE polymorphism. Harjes et al.’s (2008) conclusions were based on an association mapping panel comprised of 157 lines from temperate maize germplasm. The weaker association between LcyE-5′TE genotypes and the total proA concentration observed in this study is not surprising if we consider that several genes regulate the flux in carotenoid biosynthesis and that hydroxylation reactions also act on proA-related carotenoid components. As emphasized by Vallabhaneni et al. (2009), LcyE-5′TE is effective in controlling the pathway branching, but does not necessarily result in enhanced accumulation of proA-related compounds due to hydroxylation of proA compounds to non-proA ones.

CrtRB1 specifically controls hydroxylation of BC to BCX in maize endosperm tissues, and its alleles with reduced hydroxylation activity are associated with increased BC content and BC to BCX ratio (Vallabhaneni et al. 2009; Yan et al. 2010). We found very strong association between ‘allele 1’ of CrtRB1 and the endosperm content of BC and proA concentrations irrespective of the genotypic constitution for LcyE (Table 5 and Figure S1). Although only 4 of 15 populations H1–H15 showed strong association between ‘allele 1’ of CrtRB1 and reduction in BCX content (Table S2), this association was significant in the combined analysis across 6 digenic populations. The failure of ‘allele 1’ of CrtRB1 to significantly lower BCX content in several of the populations may be explained by a diverse regulatory system which is likely functional in maize and may involve as many as six paralogs with BC hydroxylase activities (Vallabhaneni et al. 2009), for example ZmBCH1 and ZmBCH2, which have been shown to have functional roles (Li et al. 2010). Based on chromosome location and cDNA sequences, it could be inferred that ZmBCH2 (Li et al. 2010), HYD3 (Vallabhaneni et al. 2009) and CrtRB1 (Yan et al. 2010) refer to the same locus on chr. 10.05. Li et al. (2010) reported that ZmBCH2 could only affect 5 % of BC, while ZmBCH1 (located on chr.2) could hydroxylate large amounts of both BC and BCX, when expressed transgenically in *Escherichia coli.* Besides these non-heme di-iron mono-oxygenases, the maize genome is known to have at least one copy of each P450 heme-thiolate-class BC hydroxylases, viz. CYP97A and CYP97C. This, coupled with the existence of duplicated BC hydroxylase genes elsewhere in the maize genome, suggests a complex regulatory system for maize carotenoid biosynthesis, with each hydroxylase gene playing functionally different roles. Our findings not only highlight the significance of CrtRB1 in enhancing the endosperm BC content but also suggest further possibilities to identify and manipulate other key hydroxylase genes, which may lead to achieving even higher concentrations of proA-related compounds in the maize endosperm.

Based on the positions of LcyE and CrtRB1 in the carotenoid metabolic pathway, it is reasonable to speculate that combining the mutant partial knock-out or reduced function alleles for these loci may result in higher accumulation of proA compounds than from partial knock-out of either of them alone. This hypothesis was supported by the association mapping study of Yan et al. (2010), in which a greater proportion of phenotypic variation for BC and for the ratio of BC to total carotenoid content was explained by the combined CrtRB1 and LcyE-5′TE polymorphisms than by CrtRB1 alone, although none of the inbred haplotypes in their association panels combined the most favorable polymorphisms for both loci. Association mapping can rapidly identify allelic variation for underlying genes and their interaction effects, but these findings need to be validated before embarking on large-scale marker-assisted breeding efforts.

Results from our studies to validate the effects of LcyE and CrtRB1 led to several conclusions that are useful to breeding programs using MAS for these alleles. CrtRB1 clearly has a much larger effect than LcyE on proA concentration (Fig. 2), and MAS for favorable ‘allele 1’ of CrtRB1 can lead to rapid doubling, or more, of total proA concentration. In contrast, MAS for favorable ‘allele 4’ of LcyE generally resulted in 20–30 % increase in total proA concentration. Thus, MAS for the CrtRB1 locus alone appears to be a reliable strategy for rapidly achieving genetic gains for BC and total proA carotenoids in tropical maize breeding programs.

Results for 21 populations (H1–H15 and Digenic 1–6) showed strong (20–30 %) effect of favorable ‘allele 1’ of CrtRB1 in reducing the total amount of carotenoids. We hypothesize that in the maize kernel, feedback inhibition action of upstream genes, e.g. *Psy1*, may be occurring as a consequence of the “pathway blocking” action of ‘allele 1’ of CrtRB1. Research in Arabidopsis in photosynthetic tissues has shown that the regulation of carotenoid biosynthesis is interconnected with that of related metabolic pathways as well as developmental and environmental responses where isoprenoids play a role. The expressions of all genes that encode enzymes that are known to function at each step in the carotenoid pathway are highly correlated with PSY (Meier et al. 2011; Ruiz-Sola and Rodriguez-Concepcion 2012). Another possibility is that demand for compounds downstream of zeaxanthin may be satisfied, allowing zeaxanthin to accumulate, when unfavorable ‘allele 2’ of CrtRB1 is present, whereas the diminished pool of zeaxanthin resulting from the action of ‘allele 1’ of CrtRB1 is more fully consumed by downstream reactions. Post-transcriptional regulation mechanisms including sequestration, storage capacity and carotenoid turnover have been identified to play a significant role in the carotenoid pathway, mainly in photosynthetic tissues but more research is needed in kernels (Ruiz-Sola and Rodriguez-Concepcion 2012). Nevertheless, given the fact that maximum proA was achieved, without reducing or while minimizing reduction of total carotenoid concentration, by combining homozygous favorable ‘allele 1’ of CrtRB1 with homozygous unfavorable ‘allele 2’ or heterozygous LcyE, our data suggest that breeders should avoid selecting for homozygous favorable alleles at both genes.

Our observation of SD for LcyE in all eight and for CrtRB1 in five of the eight digenic F_2_ populations is consistent with frequent observation of SD in maize and presence of many segregation distortion regions (SDRs) throughout the maize genome (Lu et al. 2002). SD in maize could be due to the presence of gametophytic factors (ga) (Mangelsdorf and Jones 1926; Neuffer et al. 1997) or to naturally occurring gene mutants like *dek* (defective kernel), *ms* (male sterile) and *emb* (embryo-specific mutation) (Neuffer et al. 1997). Besides, known *ga* genes, many SDRs have also been mapped to different regions on all 10 chromosomes of maize (Lu et al. 2002). The location of LcyE coincides with that of SDR8.2, whereas the location of CrtRB1 coincides with SDR10.2 (Lu et al. 2002). Such SD may have important implications for population sizes to be used in breeding programs. Because LcyE and CrtRB1 are co-localized with, and behave as if they are located in SDRs, and because their favorable alleles are under-represented in most of the populations studied, use of these markers should assay large numbers of segregating individuals to achieve desired numbers of favorable genotypes.

Concerns have been raised earlier that reducing the amount of carotenoids may lead to compromised abiotic stress tolerance in crop plants (Tan et al. 1997). The transcript profiling efforts for these two loci by Harjes et al. (2008) and Yan et al. (2010) revealed that the differences in expression levels were very high in endosperm, not very different in embryos, and not at all different in leaves, which suggest tissue-specific regulation of LcyE and CrtRB1. Thus selecting for mutant allele of LcyE and/or CrtRB1, whose expression is limited to endosperm is unlikely to cause any undesirable effects in the carotenoid metabolism of leaves or other vegetative tissues.

Keeping in view the current understanding of carotenoid biosynthesis in maize endosperm, efforts to attain higher proA-related compounds need to focus not only on controlling the pathway branching and carotene hydroxylation but also enhancing the total pathway flux and minimizing the carotenoid degradation (Wurtzel et al. 2012). Several genes such as phytoene synthase (Buckner et al. 1996), carotene isomerases and desaturases (Li et al. 2010) have been implicated in the overall regulation of flux in the carotenoid metabolic pathway, and genes such as *ZmCCD1* (Sun et al. 2008) and zeaxanthin epoxidases (Vallabhaneni and Wurtzel 2009) have been shown to regulate degradation of carotenoids to further downstream compounds. Current efforts at CIMMYT aim to identify and introgress variant alleles with enhanced efficiency at multiple points of the carotenoid pathway in maize, of which controlled regulation of BC hydroxylation through CrtRB1 has emerged as one of the important breeding strategies for enhanced accumulation of proA-related compounds.

## Electronic supplementary material

Below is the link to the electronic supplementary material.

Supplementary material 2 (JPG 52.7 kb)

Supplementary material 2 (DOC 115 kb)
