# Supplementary figures and images for: Validation of the effects of molecular marker polymorphisms in LcyE and CrtRB1 on provitamin A concentrations for 26 tropical maize populations
Source: Theor Appl Genet. 2012 Oct 2;126(2):389–99. doi: 10.1007/s00122-012-1987-3 (PMC3555234; doi:10.1007/s00122-012-1987-3)

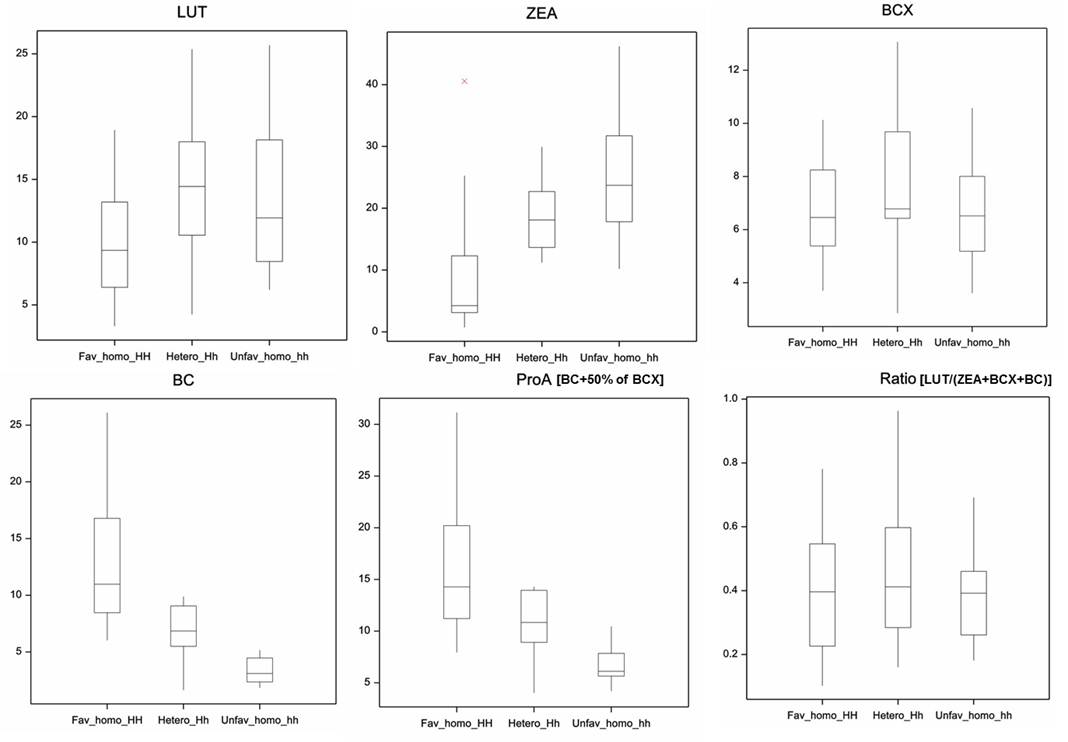

Supplement: Supplementary file 1 — Supplementary material 2 (JPG 52.7 kb) [file 122_2012_1987_MOESM1_ESM.jpg]
