# Supplementary material for: Validation of the effects of molecular marker polymorphisms in LcyE and CrtRB1 on provitamin A concentrations for 26 tropical maize populations
Source: Theor Appl Genet. 2012 Oct 2;126(2):389–99. doi: 10.1007/s00122-012-1987-3 (PMC3555234; doi:10.1007/s00122-012-1987-3)
Supplement: Supplementary file 2 — Supplementary material 2 (DOC 115 kb) [file 122_2012_1987_MOESM2_ESM.doc]

**Table S1. Pedigree information of five mono genic LcyE, 15 monogenic CrtRB1 and six di-genic (LcyE and CrtRB1) F_2_ populations used to validate the stand alone and interaction phenotypic effects**

| **Description** | **Population** | **Pedigree** |
| --- | --- | --- |
| LcyE populations | L1 | CML486/ Carotenoid Syn3-FS11-4-3-B-B-B |
|  | L2 | CML-297-B/DRB-F2-60-1-1-1-BB/[BETASYN]BC1-9-#-B |
|  | L3 | MAS[206/312]-23-2-1-1-B-B-B/[BETASYN]BC1-11-3-1-#-B/ CML297 |
|  | L4 | Hi27XCML328-F2 |
|  | L5 | P72c1xCML-297 x CL-02410-3-1-1-B/CML-297-B-B |
| CrtRB1 populations | H1 | (Carotenoid Syn3-FS8-4-3-B-B-B/(KU1409/DE3/KU1409)S2-18-2-B)-B-3 |
|  | H2 | (Carotenoid Syn3-FS8-4-3-B-B-B/(KU1409/DE3/KU1409)S2-18-2-B)-B-5 |
|  | H3 | (Carotenoid Syn3-FS8-4-3-B-B-B/(KU1409/DE3/KU1409)S2-18-2-B)-B-2 |
|  | H4 | (CML297/(KU1409/DE3/KU1409)S2-18-2-B)-B-4 |
|  | H5 | (KUI carotenoid syn-FS11-1-1-B-B-B/(KU1409/DE3/KU1409)S2-18-2-B)-B-3 |
|  | H6 | (KUI carotenoid syn-FS17-3-2-B-B-B/(KU1409/DE3/KU1409)S2-18-2-B)-B-3 |
|  | H7 | (KUI carotenoid syn-FS17-3-2-B-B-B/(KU1409/DE3/KU1409)S2-18-2-B)-B-5 |
|  | H8 | (KUI carotenoid syn-FS25-3-2-B-B-B/(KU1409/DE3/KU1409)S2-18-2-B)-B-2 |
|  | H9 | (CML297/(KU1409/DE3/KU1409)S2-18-2-B)-B-3 |
|  | H10 | (KUI carotenoid syn-FS25-3-2-B-B-B/(KU1409/DE3/KU1409)S2-18-2-B)-B-2 |
|  | H11 | (Florida A plus Syn-FS2-2-1-B-B/(KU1409/DE3/KU1409)S2-18-2-B)-B-2 |
|  | H12 | (Florida A plus Syn-FS2-2-1-B-B/(KU1409/DE3/KU1409)S2-18-2-B)-B-3 |
|  | H13 | (Florida A plus Syn-FS2-2-1-B-B/(KU1409/DE3/KU1409)S2-18-2-B)-B-4 |
|  | H14 | (Florida A plus Syn-FS2-2-1-B-B/(KU1409/DE3/KU1409)S2-18-2-B)-B-5 |
|  | H15 | (KUI carotenoid syn-FS11-1-1-B-B-B/(KU1409/DE3/KU1409)S2-18-2-B)-B-5 |
| Digenic segregating populations | Digenic-1 | (KUI carotenoid syn-FS11-1-1-B-B-B/(KU1409/DE3/KU1409)S2-18-2-B)-B† |
|  | Digenic-2 | (KUI carotenoid syn-FS17-3-2-B-B-B/(KU1409/DE3/KU1409)S2-18-2-B)-B |
|  | Digenic-3 | (KUI carotenoid syn-FS25-3-2-B-B-B/(KU1409/DE3/KU1409)S2-18-2-B)-B |
|  | Digenic-4 | (Carotenoid Syn3-FS8-4-3-B-B-B/(KU1409/DE3/KU1409)S2-18-2-B)-B |
|  | Digenic-5 | (Florida A plus Syn-FS2-2-1-B-B/(KU1409/DE3/KU1409)S2-18-2-B)-B |
|  | Digenic-6 | (CML297/(KU1409/DE3/KU1409)S2-18-2-B)-B |
|  | Digenic-7 | KUI3 X SC55 |
|  | Digenic-8 | KUI3 X B77 |

† FS=full sib; B=bulk of self-pollinations

**Table S2: Effect of CrtRB1-3’TE in the homozygous LcyE background in diverse germplasm**

| **Population** | **Genotype** | | **LUT** | **ZEA** | **BCX** | **BC** | **ProA** |
| --- | --- | --- | --- | --- | --- | --- | --- |
|  | **LycE5’TE** | **CrtRB1-3’TE** |  |  |  |  |  |
| **H1** | “2” | “2” | 6.5±0.9 | 22.5±2.4 | 10.5±0.8 | 2.2±0.3 | 7.5±0.5 |
|  | “2” | “1” | 9.3±1.2 | 3.4±0.6 | 4.1±0.5 | 9.1±1.1 | 11.1±0.9 |
|  | “2” | “H” | 10.8±1.8 | 13.3±1.7 | 6.5±0.4 | 6.5±1.3 | 9.8±0.9 |
| **H2** | “2” | “2” | 6.9±0.6 | 10.2±1.1 | 4.0±0.7 | 2.1±0.5 | 4.2±1.1 |
|  | “2” | “1” | 11.2±1.5 | 1.2±0.3 | 5.0±0.5 | 12.7±1.4 | 15.2±1.1 |
|  | “2” | “H” | 22.3±2.1 | 19.4±1.6 | 8.5±0.3 | 9.9±0.9 | 14.1±0.7 |
| **H3** | “2” | “2” | 6.2±1.1 | 11.9±2.1 | 5.1±1.1 | 1.8±1.2 | 4.4±1.2 |
|  | “2” | “1” | 6.2±0.8 | 0.7±0.1 | 3.7±0.9 | 9.2±0.9 | 11.0±0.9 |
|  | “2” | “H” | 14.4±1.3 | 17.5±1.9 | 6.9±0.8 | 3.9±1.1 | 7.4±1.0 |
| **H4** | “2” | “2” | 15.6±1.8 | 16.6±1.8 | 6.3±0.9 | 2.7±0.3 | 5.9±0.5 |
|  | “2” | “1” | 8.5±0.9 | 3.4±0.5 | 6.6±1.1 | 10.7±1.2 | 14.0±1.2 |
| **H5** | “2” | “2” | 12.0±1.1 | 18.9±1.5 | 3.6±0.9 | 4.1±0.7 | 6.0±0.8 |
|  | “2” | “1” | 18.8±2.3 | 5.6±0.7 | 6.0±0.6 | 12.4±1.3 | 15.5±1.2 |
|  | “2” | “H” | 21.0±3.2 | 15.9±1.1 | 2.8±0.5 | 3.0±0.6 | 4.4±0.6 |
| **H6** | “2” | “2” | 9.5±0.8 | 27.1±2.1 | 7.3±0.5 | 2.5±1.2 | 6.2±1.1 |
|  | “2” | “1” | 6.5±0.6 | 4.3±0.5 | 7.5±0.6 | 18.9±0.9 | 22.7±0.7 |
|  | “2” | “H” | 14.8±1.1 | 11.2±1.6 | 6.7±1.2 | 6.0±1.1 | 9.3±1.1 |
| **H7** | “2” | “2” | 11.8±1.2 | 20.4±1.7 | 7.1±0.9 | 2.2±0.8 | 5.8±0.9 |
|  | “2” | “1” | 11.9±1.5 | 5.7±0.8 | 9.5±1.2 | 22.9±1.8 | 27.7±1.6 |
|  | “2” | “H” | 7.0±0.8 | 12.5±1.8 | 10.0±1.4 | 8.9±0.9 | 13.9±1.2 |
| **H8** | “2” | “2” | 15.5±1.6 | 43.1±2.8 | 10.5±1.9 | 5.1±1.2 | 10.4±1.5 |
|  | “2” | “1” | 17.6±1.4 | 9.9±1.8 | 10.1±1.1 | 26.1±3.4 | 31.1±2.8 |
|  | “2” | “H” | 25.3±1.8 | 18.1±0.9 | 13.0±0.8 | 6.8±0.8 | 13.3±0.8 |
| **H9** | “2” | “2” | 17.2±2.3 | 26.2±2.1 | 6.8±0.9 | 3.7±0.8 | 7.1±0.9 |
|  | “2” | “1” | 12.8±1.8 | 3.4±0.6 | 6.1±1.2 | 14.6±1.2 | 17.6±1.2 |
|  | “4” | “1” | 4.2±0.8 | 2.7±0.4 | 5.6±1.5 | 6.7±0.9 | 9.6±1.1 |
|  | “H” | “2” | 19.7±1.5 | 20.7±1.3 | 5.2±0.8 | 2.4±0.5 | 5.0±0.6 |
| **H10** | “4” | “2” | 22.2±2.4 | 46.2±3.4 | 6.6±0.9 | 4.4±0.5 | 7.7±0.7 |
|  | “4” | “1” | 3.3±0.7 | 4.1±0.7 | 6.8±0.8 | 21.3±1.3 | 24.7±1.1 |
|  | “4” | “H” | 4.2±0.5 | 13.7±1.5 | 6.4±1.3 | 6.4±1.1 | 9.6±1.4 |
| **H11** | “4” | “2” | 25.7±1.8 | 31.9±1.9 | 4.9±0.4 | 3.4±1.1 | 5.9±0.9 |
|  | “4” | “1” | 5.3±1.0 | 14.6±2.4 | 7.0±0.9 | 7.7±0.9 | 11.3±0.9 |
|  | “4” | “H” | 12.9±1.6 | 21.6±2.3 | 6.7±1.1 | 9.5±0.9 | 12.8±1.0 |
| **H12** | “4” | “2” | 8.4±1.2 | 31.5±2.7 | 10.3±1.1 | 4.5±1.1 | 9.7±1.1 |
|  | “4” | “1” | 18.9±1.9 | 19.2±1.8 | 3.8±0.2 | 6.0±0.8 | 7.9±0.6 |
|  | “4” | “H” | 9.5±1.5 | 21.6±2.3 | 10.4±0.3 | 8.7±0.6 | 13.9±0.5 |
| **H13** | “4” | “2” | 8.7±0.9 | 24.8±1.9 | 8.6±0.8 | 4.9±0.6 | 9.3±0.7 |
|  | “4” | “1” | 9.3±1.4 | 25.2±2.1 | 8.9±0.9 | 9.7±0.9 | 14.1±0.9 |
|  | “4” | “H” | 13.2±1.4 | 29.9±3.1 | 6.4±1.3 | 7.6±1.1 | 10.8±1.2 |
| **H14** | “4” | “2” | 19.0±1.7 | 44.7±2.9 | 6.1±0.6 | 4.9±1.3 | 7.9±1.2 |
|  | “4” | “1” | 13.5±0.8 | 40.5±3.2 | 9.5±0.7 | 6.4±1.2 | 11.2±1.1 |
|  | “4” | “H” | 15.1±0.8 | 29.0±3.1 | 9.5±0.8 | 9.5±1.5 | 14.3±1.4 |
| **H15** | “H” | “2” | 8.4±1.3 | 12.7±1.4 | 6.1±0.8 | 2.4±0.4 | 5.4±0.6 |
|  | “H” | “1” | 8.8±0.9 | 2.4±0.4 | 6.2±0.1 | 11.2±1.5 | 14.3±0.8 |
|  | “H” | “H” | 16.9±1.1 | 25.7±1.8 | 4.7±0.2 | 1.6±0.4 | 4.0±0.3 |

LUT: Lutein, ZEA: Zeaxanthin, BCX: Betacryptopxanthin, BC: Beta-carotene, ProA : Total Provitamin A carotenoids, Ratio: Ratio of α- to β-branch carotenoids

Figure S1. Effect of CrtRB1-3’TE on different carotenoid components based on 15 populations, representing diverse tropical genetic backgrounds
